# Electrical and Mechanical Ventricular Activation During Left Bundle Branch Block and Resynchronization

**DOI:** 10.1007/s12265-012-9351-1

**Published:** 2012-02-07

**Authors:** Marc Strik, François Regoli, Angelo Auricchio, Frits Prinzen

**Affiliations:** 1 Department of Physiology, Cardiovascular Research Institute Maastricht, Maastricht University, Maastricht, 6200 MD the Netherlands; 2 Fondazione Cardiocentro Ticino, Lugano Ticino, Switzerland; 3 Department of Physiology, Maastricht University, P.O. Box 616, 6200 MD Maastricht, the Netherlands

**Keywords:** Left bundle branch block, Cardiac resynchronization therapy, Electrical mapping

## Abstract

Cardiac resynchronization therapy (CRT) aims to treat selected heart failure patients suffering from conduction abnormalities with left bundle branch block (LBBB) as the culprit disease. LBBB remained largely underinvestigated until it became apparent that the amount of response to CRT was heterogeneous and that the therapy and underlying pathology were thus incompletely understood. In this review, current knowledge concerning activation in LBBB and during biventricular pacing will be explored and applied to current CRT practice, highlighting novel ways to better measure and treat the electrical substrate.

## Introduction

In the past decade, cardiac resynchronization therapy (CRT) has become an important treatment option for symptomatic heart failure patients with reduced left ventricular (LV) ejection fraction and abnormal QRS duration mostly in the form of left bundle branch block (LBBB). Until recently, little was known about the pathophysiology of the conduction disease and its adverse effects on ventricular contractility. Large clinical trials have shown that CRT benefits most patients but approximately one third of patients to not show clinical or echocardiographic response. Furthermore, further analysis of these studies show that the amount of responders was considerably higher in patients with LBBB as compared with patients without this conduction disease [1]. These aspects have revamped major interest in LBBB. This interest is further enforced by the difficulties of echocardiographic mechanical dyssynchrony measurements to adequately predict CRT response. Insight in electrical activation during LBBB and ventricular pacing during CRT has steadily increased over the last few years. LBBB appears to be the hallmark conduction disease that is treatable by CRT independently from etiology, as evidenced by efficacy of CRT in canine hearts with isolated LBBB and in CRT patients with LBBB compared to CRT patients with other conduction disorders [2, 3]. It is also becoming increasingly apparent that the site of LV stimulation, the electrical and mechanical activation patterns, and the presence of little or no scar are all critical for CRT success. In this paper, electrical and mechanical activation patterns during LBBB and CRT are discussed. Why these aspects are important for the delivery of a successful therapy is also shown.

## Left Bundle Branch Block

One hundred years ago, Eppinger and Tothberger reported distinctive changes in QRS morphology after the destruction of only a small region in the interventricular septum in canine hearts [4]. Since the esophageal-to-rectal leads in the dogs were directly extrapolated to leads II and III in human patients, LBBB too was erroneously diagnosed as right bundle branch block (RBBB) and vice versa for 25 years. This misinterpretation illustrates the fact that the conduction disease was not considered important, and LBBB was solely considered a sign of poor prognosis. Only later it was discovered that most patients with LBBB who died shortly after diagnosis died of underlying heart disease and that the conduction disease on its own was not as dangerous as previously believed. It was not until 1972 that the anatomy of the left bundle branch (LBB) was described in more detail. A histopathological study in human patients without known cardiac disease, showed that the LBB is a continuation of the His bundle and initiates between the non-coronary and right-coronary aortic cusps. It runs as a 6 to 10-mm wide ribbon-like structure under the septal endocardium in inferior and anterior directions [5]. The fibers of the LBB fibers then separate to form fasciculi into anterior, posterior, and often septal radiations in heterogeneous patterns. Ultimately, the peripheral Purkinje fibers are coupled with individual (sub)endocardial myocardial cells which allows fast depolarization of the LV [6]. In isolated human hearts with an intact LBB, extensive electrical mapping showed up to three LV endocardial breakthrough sites which resulted in a rapid electrical activation of the LV [7].

**Fig. 1:**
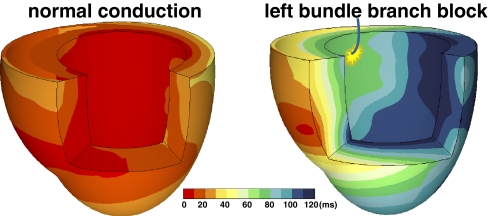
Typical examples of 3D electrical activation in canine hearts during normal conduction (*left panel*) and after creation of left bundle branch block (*right panel*). Each electrical activation map is reconstructed using a single-beat recording of simultaneous epicardial and endocardial electrical mapping. Epicardial potentials were derived using electrode bands placed around the heart, containing over 100 contact electrodes while the LV endocardium was mapped using custom-made plunge electrodes [63]. Early activated regions are indicated by a *red color* (close to 0 ms) and late activation regions are indicated by a *dark blue* color (over 100 ms), see color bar. Reproduced with permission [8]

**Fig. 2:**
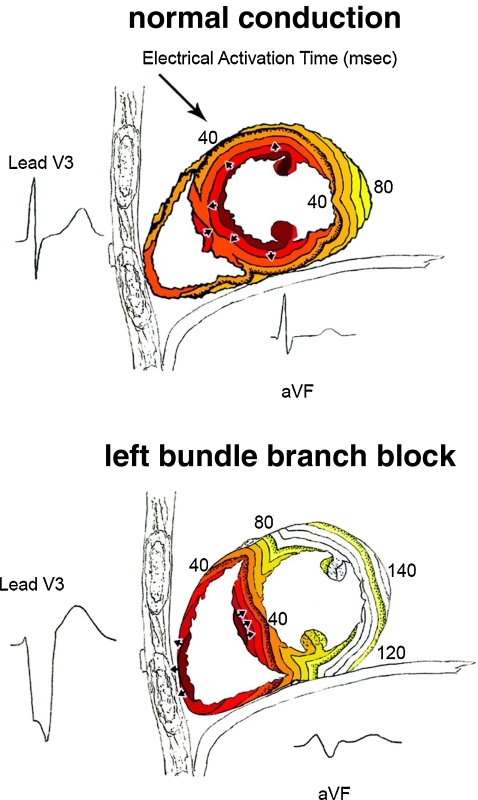
Timing of electrical activation (depolarization) wavefronts in normal conduction and during LBBB shown in sagittal view. For reference, two QRS-T waveforms are shown in their anatomic locations (V3 on the chest and aVF inferiorly). Electrical activation starts at the *small arrows* and spreads in a wavefront with each colored line representing successive 10 ms. In normal conduction, activation begins within both the LV and RV endocardium. In LBBB, activation only begins in the RV and must proceed through the septum before reaching the LV endocardium. Reproduced with permission [64]

**Fig. 3:**
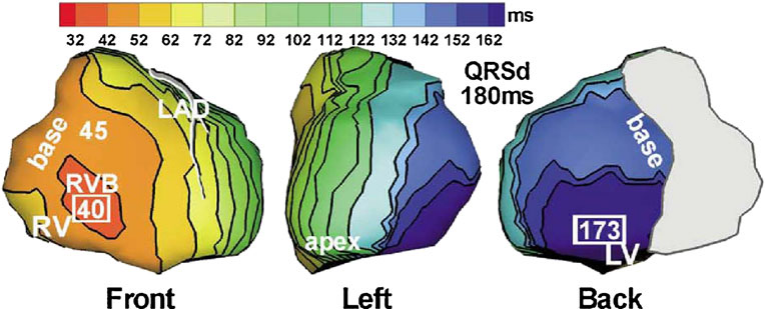
Epicardial isochrone maps during native rhythm in a patient with LBBB. Left anterior escending (LAD) coronary artery is shown and the approximate valve region is covered by gray. Earliest and latest ventricular activation times (in milliseconds) are indicated by *framed numbers*. Activation times are given with respect to QRS onset. *QRSd* QRS duration. Reproduced with permission [10]

Investigation of the electrical activation during LBBB can be performed in patients but is limited by the presence of comorbidities and the lack of knowledge concerning the duration and extent of the lesion (or lesions). Animal models can be used to specifically investigate the effects of *isolated* LBBB as discussed more extensively elsewhere in this edition [8]. Examples of three-dimensional activation time maps in a canine heart before and after induction of LBBB are shown in Fig. 1. In LBBB, onset of electrical activation occurs inside the right ventricle and the electrical wavefront then slowly propagates through the interventricular septum towards the lateral wall of the LV [9]. As is also shown in a schematic representation of transmural conduction during normal and LBBB conduction in humans (Fig. 2), LBBB reverses transseptal activation and causes large changes in QRS morphology and duration. Electrocardiographic imaging (ECGi, a novel technique that extracts estimated epicardial activation sequence from body surface maps) shows multiple epicardial conduction patterns in CRT candidates with LBBB of which an example is shown in Fig. 3 [10]. The LV endocardial activation sequence during LBBB has also proven to be heterogeneous in heart failure patients as shown by conventional point-by-point technique or three-dimensional electroanatomical reconstruction contact (Carto) and non-contact mapping (EnSite) technique. These studies have shown that in LBBB patients with heart failure, LV endocardial breakthrough is heterogenous and may occur at different septal regions [11, 12]. In some patients, breakthrough occurred in the mid-septal region, which could suggest activation by slow conduction through the LBB, in some others, via right-to-left transseptal activation as observed in dog models [11]. Narula reported in 1977 that by distal His-Bundle pacing in the right ventricle, he was able to abolish the electrocardiographic signs of LBBB in 25 patients, thereby “curing” the conduction disease. In these LBBB patients, the lesions were apparently located proximal in the rapid conduction system, just below the AV node [13].

The varying conduction patterns could thus be caused by one or more of the following aspects: (1) the varying structure of the LBB, (2) variability in the location of the LBB “block”, being either a proximal lesion or a more distal and diffuse disease, (3) LV hypertrophy and fibrosis as associated cellular uncoupling can result in increasing QRS duration and a LBBB-like QRS morphology [14]. The heterogeneous activation patterns seen in heart failure patients with LBBB might in part explain why CRT leads to varying amount of response. In addition, mentioned data accentuate the need for patient tailored therapy by carefully selecting the site of pacing and pacing settings.

### Electrophysiological Evaluation of the CRT Candidate

The key clinical investigation to detect and evaluate the extent of ventricular conduction delay remains the surface electrocardiogram. To diagnose LBBB in patients, specific ECG criteria exist in addition to QRS width ≥120 ms such as a broad notched or slurred R wave in leads I, aVL, V5, and V6, an occasional RS pattern in V5 and V6 attributed to displaced transition of QRS complex, and absent q waves in leads I, V5, and V6 (in the absence of a large anterior-apical infarction) [15]. Note that although scar in the septum causes q waves in V1 to V3 when normal conduction is present, the same scar causes large R waves in V1 to V3 in the presence of LBBB because of unopposed electrical forces in the RV free wall [16]. When these criteria are not met, it is likely that patients have RBBB or slowed conduction by LV hypertrophy. Some heart failure patients with RBBB have been shown to have LV conduction delay similar to LBBB patients [17] but large trials have failed to show CRT response in these patients [18–20]. Recently, it has been shown that patients with QRS duration >150 ms and LBBB morphology show the highest response rate in large multicenter trials [21, 22]. It should be mentioned however that in most CRT trials, patients were required to have QRS duration of at least 120 ms, and approximately one third of these patients did not have LBBB [3, 20]. On top of that, one third of patients diagnosed with LBBB by conventional electrocardiographic criteria may not have true complete LBBB but likely have a combination of left ventricular hypertrophy and left anterior fascicular block [9, 23]. A recent electrical mapping study showed that “true LBBB” was only seen in patients with a QRS duration exceeding 140 ms [24].

In the past decade, most efforts focused on finding dyssynchrony parameters to predict CRT response were based on echocardiography, which are discussed later in this review. Interestingly, recent studies have revamped the interest in using the surface ECG for exactly that purpose. An example is the PROSPECT trial where multiple echocardiography derived dyssynchrony parameters were investigated, followed by a sub-study where various ECG parameters were tested [22, 25]. In fact, LBBB morphology was the only parameter that was predictive for both volumetric and clinical response after 6 months (defined by LV end-systolic volume reduction of ≥15% and improvement in Clinical Composite Score, respectively). In addition, a broad QRS complex during single-site LV pacing was predictive of failure of volumetric response (OR = 0.86 per 10 ms increment). In non-ischemic patients, an LV paced QRS width of ≤200 ms was five times more likely to be associated with a positive response than having a LV paced QRS width greater than 200 ms, while no such difference was found in ischemic patients. The authors of this article postulate that LV paced QRS width may be an indirect method of identifying a region near scar or an area of poor conduction. Sweeney et al. [26] carefully inspected standard 12-lead electrocardiograms of 202 LBBB patients indicated for CRT. Based on the comparisons of baseline and post-implant electrocardiograms the authors introduced new measurements, which predicted CRT response (defined as at least 10% reduction in end-systolic volume as derived by echocardiography at 6 months). A notch, which occurred after 40 ms of QRS onset, was regarded as the transition from RV to LV depolarization and the time difference between this notch and the end of QRS was indicated as the LV activation time (LVAT_max_). QRS duration was weakly associated with reverse remodeling probability and this relationship was replaced by LVAT_max_ in the multivariable model. A longer LVAT_max_ at baseline was predictive of CRT response (OR 1.30 for each 10 ms increase up to 125, *p* = 0.001). The Selvester QRS score was used to quantify LV scar and a higher score was detrimental to volumetric response (OR 0.49 for each 1 point increase from 0 to 4, *p* = 0.002) [24]. The appearance of anterior forces in the precordial leads after implantation (change in R amplitude in *V*_1_ and *V*_2_ in expected direction) was also predictive of CRT response. An alternative method to estimate LV electrical asynchrony is by calculating the delay between QRS onset and LV lead depolarization. Varma found in heart failure patients that this delay exceeded 100 ms in 87% of LBBB patients as compared to 45% of RBBB patients, even though there was no difference in QRS duration [27]. Singh et al. [28] showed that CRT patients with a reduced LV lead electrical delay (<50% of the QRS duration) before biventricular pacing was associated with worse clinical outcome at 12 months.

Studies investigating electrocardiography beyond surface ECG or pacemaker lead electrograms are even scarcer because evaluation of cardiac electrical activation sequence by catheter mapping in CRT candidates is time-consuming, cumbersome, and not without risk. Lines of conduction block are seen in most LBBB patients as shown by endocardial (EnSite) and epicardial (ECGi) non-contact mapping studies [10, 12]. The implications of these lines of block have been investigated in a small observational study where non-contact mapping was performed in 23 CRT candidates [29]. Twelve of the 18 patients who had lines of conduction block before implantation were volumetric CRT responders at 3 months as opposed to one of the eight patients who had homogeneous endocardial conduction (*p* = 0.01). This study confirmed that the benefit of CRT is more dependent on specific LV activation patterns rather than on total LV activation time, which could explain why LVAT_max_ beyond 125 ms, and in some studies QRS duration, are poor individual predictors of response [26, 30].

### Mechanical Evaluation of the CRT Candidate

Although the underlying pathophysiological mechanisms of how LBBB induces left ventricular dysfunction remain to be clarified, LBBB yields a disturbance of mechanical coordination between the different regions of the left ventricular walls, especially between septum and lateral wall. Moreover, the extremely variable and relatively unpredictable nature of response to CRT remains puzzling. Transthoracic echocardiography represents a non-invasive method that has been used to address some of these issues. The first measurements such as interventricular mechanical delay, septal posterior wall motion delay and pulsed-wave, color-coded tissue Doppler imaging were all measures of myocardial regional mechanical delay in patients with heart failure and ventricular conduction delays (mostly LBBB, Table 1) [25, 31, 32]. The very promising initial findings derived mainly from single center studies, showing excellent predictive value of these parameters were offset by the more recent results of the PROSPECT and J-CRT trials [25, 33]. In these trials, area under receiver-operating characteristics curve (ROC) for positive clinical or volume response after CRT was ≤0.62. Possible reasons accounting for the shortcomings of these measures include inability to discern passive from active wall movements, limited 2-D plane and operator dependency. However, some studies showed that timing parameters as determined by the gold-standard deformation technique of MRI tagging were also not able to predict CRT response, indicating that timing parameters may have intrinsic limitations [34].

**Table 1:** Most accurate (sensitivity > 80%, specificity > 75%, or ROC > 0.80) measurements to detect mechanical discoordination caused by ventricular conduction delay, such as LBBB, in CRT candidates

Author	Parameter	Design	Patients (*n*)	Ischemic etiology (%)	Follow-up (months)	Cut-off
Echocardiographic measurements of regional delay						
Soliman (2009) [57]	3D-SDI	SC, obs	90	51	1	>10%
Bax (2004) [58]	Ts-4	SC, obs	80	55	6	65 ms
Yu (2003) [59]	Ts-SDI2	SC, obs	30	40	3	32.6 ms
Suffoletto (2006) [60]	2D-RS	SC, obs	50	62	8	130 ms
Echocardiographic measurements of mechanical discoordination and inefficiency						
Jansen (2007) [61]	Shuffle and septal motion	SC, obs	53	49	3	NA
Buss (2009) [62]	EPI	SC, obs	42	43	6–8	59%
De Boeck (2009) [40]	SRSsept	SC, obs	62	44	6.5	4.7%
Lim (2008) [37]	Strain-delay	SC, obs	62	35	3	25%
Cardiac magnetic resonance measures of dyssynchrony						
Bilchick (2008) [42]	CURE index	SC, obs, control	20	40	/	< 0.75

*2d-RS* speckle tracking radial strain; *3D-SDI* standard deviation of 16 time-volume peaks; *CURE index* circumferential uniformity ratio estimate; *EPI* echocardiographic phase imaging; *obs* observational; *SC* single center; *SRS Sept* systolic rebound stretch in the septum; *Ts-4* maximal velocity delay between four basal segments; *Ts-SD12* standard deviation of velocity peaks in 12 basal and midventricular segments

Further development of echocardiographic methodologies coupled with the advances in the field of cardiac magnetic resonance have provided greater depth of understanding in the complex mechanical myocardial wall behavior during LBBB. Some additional aspects of the physiology of myocardium wall motion during LBBB should be taken into account. Myocardial fiber spatial disposition in three different layers cause the mechanical contractile movement to be nonsymmetrical and in different directions. Myocardial contractile thickening has been observed to be longitudinal, circumferential, and radial, thus conferring a movement of torsion during systole [35]. In the context of important ventricular conduction delay such as LBBB, myocardial regional motion is characterized by complex and multiphasic mechanical behavior [36]. It follows therefore that measures evaluating global wall motion or myocardial efficiency in all three different perspectives, rather than regional two-dimensional measures, may be more suitable to characterize contractile mechanics during LBBB. With these notions, echocardiography has manifested a new drive in the last few years with the emergence of new dyssynchrony measures. The most widely used technique for the detection of myocardial deformation by discriminating active from passive wall motion is speckle tracking imaging (STI) strain. By considering the sum of the difference between peak and end-systolic strain across 16 segments (strain delay index), Lim et al. [37] found that longitudinal strain by STI in HF patients with ventricular conduction delay was predictive of CRT response. Another study by Klimusina et al. [38] utilized segmental peak myocardial strain in heart failure patients derived from speckle tracking and showed that heart failure patients with intraventricular conduction delay presented heterogeneity in longitudinal and radial strain distributions, with amplitudes being particularly low in the septum and higher in the lateral and posterior walls. Based on the Speckle Tracking and Resynchronization Study (STAR) for prediction of volume response after CRT, ROC for circumferential and longitudinal speckle-tracking was 0.59 and 0.57, respectively, whereas that of radial and transverse strain was superior (0.79 and 0.75, respectively). It should be mentioned however that a proportion of patients without detected dyssynchrony nevertheless showed reverse remodeling after CRT; these patients were most likely to present a wide QRS and non-ischemic heart failure etiology [39]. One of the most promising echocardiographic indices is Septal Rebound Stretch (SRSsept). SRSsept is defined as systolic stretch occurring after initial shortening in the early activated septum. De Boeck et al. [40] showed that SRSsept strongly correlated with an increase in LV ejection fraction after CRT as well as a reduction in LVESV and brain natriuretic peptide. SRSsept predicted a 15% reduction in LVESV with an AUC of 0.81.

Cardiac magnetic resonance is a high resolution image modality which allows complex and global assessment not only of cardiac morphology, but also function. Indeed, this imaging modality has emerged to become the “gold-standard” for the characterization of myocardial contractile behavior. By using MRI tagging, time plots of strain are generated for each segment in each short-axis slice, and the circumferential uniformity ratio estimate (CURE) is calculated [41]. This ratio considers “0” most dyssynchronous and “1” absence of dyssynchrony. A CURE cut-off of <0.75 accurately predicts symptomatic response after CRT [42]. An important additional aspect is that CURE uses information from the full cardiac cycle and not only time-to-peak. The paucity of consistent prospective, multicenter data on echocardiographic and CMR parameters estimating LV dyssynchrony during LBBB, along with the expensive, time-consuming, and knowledge-intensive nature of these investigations, have limited the diffusion of such technologies to select the adequate CRT candidate.

### CRT in the Ischemic Dyssynchronous Heart

**Fig. 4:**
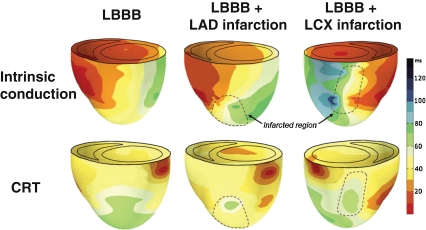
3D reconstruction of electrical activation times of the LV and the RV during intrinsic conduction (LBBB) and BiV pacing (at the RV apex and basal-lateral LV wall) in representative hearts with LBBB (*left*), LBBB with LAD infarction (*middle*), and LBBB with LCX infarction (*right*). Reproduced with permission [65]

Most studies show that CRT response is higher in non-ischemic patients [43, 44]. A possible mechanism lies in modification of the electrical substrate. According to this idea, the extent of resynchronization would be limited as a result of slow-conducting or non-conducting regions. This would mean that a good response to CRT not only requires clear conduction disease, but also the capability to properly resynchronize the heart. Figure 4 shows examples of how an LAD or LCX infarction can influence electrical resynchronization in dogs with LBBB. An important feature in this regard is the site of pacing as pacing in the vicinity of scar tissue can compromise conduction. In canine hearts with LBBB and transmural infarction, pacing away from the infarcted regions resulted in a similar CRT response as in non-infarcted canine LBBB hearts [45].

In line with the pre-clinical data, clinical response after CRT is assumed to involve three key factors: lead position (in relation to area of greatest delay or vicinity to scar tissue), the presence of discoordinate and inefficient area of myocardial contractility, and quantity/degree of scarred tissue. However, rapid, effective, and integrated assessment of these features in the diagnostic build-up of patients considered for CRT, remains an unresolved challenge. Furthermore, the present literature offering aid remains scanty. Some important single center studies have shown how measures derived from CMR may be of great use. The relation between scar and left ventricular lead deployment has been unveiled using CMR late gadolinium enhancement [46, 47]. This method, besides emphasizing how scar size and transmurality were associated to a dismal prognosis after CRT, also showed how pacing scar localized in a postero-lateral area reduces CRT response rate. Another CMR study, which examined the relationship between left ventricular myocardial dyssynchrony (circumferential uniformity ratio estimate derived from myocardial tagging—MT) and scar location (delayed enhancement—DE), found that functional class improvement after CRT was predicted by MT and that DE offered further predictive value [42]. Though the data are limited, there is no doubt that the development of integrated measures derived from CMR may be of great use to improve selection eligible for CRT (see Table 1).

**Fig. 5:**
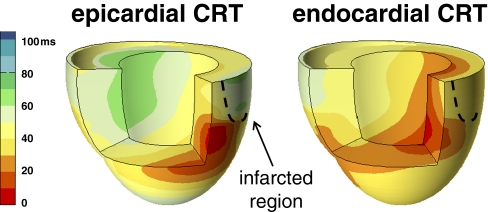
Typical examples of 3D electrical activation in canine hearts with chronic LBBB and transmural myocardial infarction during CRT with epicardial LV pacing (*left panel*) and endocardial LV pacing (*right panel*). Reproduced with permission [1]

Worthy to mention is the study by Delgado et al. [48] who have addressed the relative influence of ventricular dyssynchrony, left ventricular lead position, and myocardial scar on the long-term prognosis of patients treated with CRT. Through the use of two-dimensional speckle tracking radial strain imaging LV dyssynchrony (difference between the earliest and latest of six segments) was measured and the regions of scar were identified (considered segments with peak radial strain value <16.5%). After CRT, the influence of LV lead position was assessed retrospectively radiographically and placed in relation to the segment of greatest delay or the area of scar. The investigators found that the independent predictors of unfavorable prognosis after CRT were discordant LV lead position with respect to the radial segment of greatest delay and the presence of myocardial scar at the level of the pacing lead. A significantly better prognosis was found in patients with a substantial time delay between the antero-septal and posterior segments (≥130 ms). In spite of the methodological limits of the study, i.e., only radial strain to assess left ventricular dyssynchrony [49] and simple retrospective radiographical assessment of lead position, this study is the first which demonstrates how lead position, electro-mechanical delay, and presence of scar are inextricably linked and impact on the prognosis of CRT patients. Leyva et al. [47] also showed that LV lead position inside the scar region results in a fivefold risk of pump failure and sudden cardiac death as compared to patients with no scar and patients with scar, but LV lead positioned elsewhere combined. Besides avoiding scar tissue, the position of the LV could also be improved by moving from the epicardium (epicardial implant or coronary sinus) to the endocardium. Under physiological conditions, excitation of the LV initiates at the endocardium [7] while in CRT, the LV is most often paced at the epicardial surface. As discussed, conventional CRT can resynchronize the heart but not to (near) normal conditions. In canine LBBB hearts with myocardial infarction, endocardial LV pacing during CRT consistently improved systolic LV pump function, reduced electrical dyssynchrony and decreased dispersion of repolarization, as compared to epicardial LV pacing at the same site (Fig. 5) [50]. Additionally, the hemodynamic effects for endocardial sites were less dependent on location and AV-delay than epicardial sites. The added benefit of endocardial CRT was also seen in dogs with heart failure (induced by tachypacing) [50] and in dogs with isolated LBBB [51]. Support for these experimental findings come from studies by Spragg et al. [52] in seven ICM patients where LV endocardial and epicardial pacing at immediately transmural sites gave equivalent LV dP/dt_max_ values. However, LV dP/dt_max_ at best LV endocardial sites was greater than conventional CRT. Given individual variations in etiology, severity, patterns of delayed ventricular activation, location of regions of scar, and extent of mitral regurgitation in heart failure, it seems indeed unlikely that one pacing site will “fit all”. Individual tailoring of endocardial CRT by searching the optimal pacing site within the endocardium is warranted. LV endocardial pacing in humans can be established through an atrial transseptal approach [53, 54] or a left transapical approach can be used [55]. In the future, wireless endocardial LV stimulation [56] might become the most feasible approach.

## Conclusions

Left bundle branch block results in asynchronous electrical activation, which can be largely reversed by CRT, thus conferring favorable clinical effects. It is imperative that correct electrical substrate, preferably in the form of *true* left bundle branch block, coexists with adequate morphological and anatomical conditions to allow adequate CRT delivery. In the future, integrated multi-modality imaging presents the central and challenging task to transfer pre-clinical knowledge into clinical practice, hence further defining the complex mechanical and functional effects of LBBB. On the other hand, with the aid of endocardial LV pacing, with- or without RV pacing, patient-specific tailoring of CRT will likely increase therapy success.
